# Effective web-based clinical practice guidelines resources: recommendations from a mixed methods usability study

**DOI:** 10.1186/s12875-023-01974-1

**Published:** 2023-01-24

**Authors:** Wei Wang, Dorothy Choi, Catherine H. Yu

**Affiliations:** 1 Department of Medicine, University of Toronto, 1 King’s College Cir, Toronto, ON M5S 1A8 Canada; 2 Li Ka Shing Knowledge Institute of St. Michael’s Hospital (Unity Health Toronto), 30 Bond Street, Toronto, ON M5B 1W8 Canada

**Keywords:** Clinical practice guidelines, Usability, Website creation, Diabetes, Digital communication

## Abstract

**Background:**

Clinical practice guidelines (CPG) are an important knowledge translation resource to help clinicians stay up to date about relevant clinical knowledge. Effective communication of guidelines, including format, facilitates its implementation. Despite the digitalization of healthcare, there is little literature to guide CPG website creation for effective dissemination and implementation. Our aim was to assess the effectiveness of the content and format of the Diabetes Canada CPG website, and use our results to inform recommendations for other CPG websites.

**Methods:**

Fourteen clinicians (family physicians, nurses, pharmacists, and dieticians) in diabetes care across Canada participated in this mixed-methods study (questionnaires, usability testing and interviews). Participants “thought-aloud” while completing eight usability tasks on the CPG website. Outcomes included task success rate, completion time, click per tasks, resource used, paths, search attempts and success rate, and error types. Participants were then interviewed.

**Results:**

The Diabetes Canada CPG website was found to be usable. Participants had a high task success rate of 79% for all tasks and used 144 (standard deviation (SD) = 152) seconds and 4.6 (SD = 3.9) clicks per task. Interactive tools were most frequently used compared to full guidelines and static tools. Misinterpretation accounted for 48% of usability errors. Participants overall found the website intuitive, with effective content and design elements.

**Conclusion:**

Different versions of CPG information (e.g. interactive tools, quick reference guide, static tools) can help answer clinical questions more quickly. Effective web design should be assessed during CPG website creation for effective guideline dissemination and implementation.

**Supplementary Information:**

The online version contains supplementary material available at 10.1186/s12875-023-01974-1.

## Background

Clinicians require up-to-date and reliable resources to keep up their knowledge. Clinical practice guidelines (CPGs) are an important knowledge translation tool that can address this need, but uptake of CPGs can be inconsistent [1, 2]. Previous research has identified that aside from content itself, effective communication is also required to improve guideline implementation [2, 3]. Format of CPGs, for example, is identified as one of 6 key domains of implementability by Kastner and colleagues [3]. However, less than half of available guideline assessment tools address evaluating the format of guidelines [4]. Notably, even in tools that do evaluate format such as GLIA and CAN-IMPLEMENT, the focus is on the formatting of content within a document e.g. using bold text for recommendations or including visual algorithms [5, 6]. These recommendations do not address the full scope of formatting considerations, since many CPGs are more versatile now than a single document.

Increasingly, guideline organizations are creating multiple versions of their CPGs, which has been thought to improve guideline dissemination [7]. Short summary versions of guidelines and interactive tools based on guideline content may be utilized more easily at the bedside than the full text version. The website modality is particularly suited for keeping these different versions of content in one accessible location. As the internet is already starting to replace traditional sources of information like books and colleagues, CPG websites will only become more common [8, 9]. However, to our knowledge, none of the current guideline assessment tools make recommendations specific to CPG website creation.

We performed a mixed methods usability study of the Diabetes Canada (DC) CPG website, a comprehensive CPG website with a wide reach to both clinicians and the general public [10–14]. Usability testing has been widely used to assess and improve health technologies including websites [12]. Our aim was to assess the effectiveness of the content and format of DC Guidelines website, using our results to inform recommendations for this and other CPG websites.

## Methods

We used mixed methods consisting of (a) questionnaire, (b) usability testing, and (c) semi-structured interviews to assess the live version of the Diabetes Canada Clinical Practice Guidelines website, updated April 10, 2018 [10].

### Website

The Diabetes Canada CPG website has a wide reach with health care providers (HCPs) as well as the general population [11]. The content of the website can be divided into 4 categories. Its contents and main components are described in Tables 1 and 2.

**Table 1:** Content of the DC Guidelines Website

*Full Guidelines*	Full text of the CPGs, separated by chapter per webpage, with download links to the portal document format (PDF) of the chapters
*Quick Reference Guide*	A 10-page abridged summary of the full CPGs containing information thought to be most relevant to providers, accessed as a PDF.
*Interactive Tools/Clinical Decision Support Tools*	Interactive tools, in which user data input generates different outputs, in line with the recommendations of the CPG text. For example, the “Self-Monitoring of Blood Glucose” (SMBG) tool asks for input from the user on the type of diabetes a patient has, their A1C, and their current treatments. The output is a recommendation for the frequency of SMBG monitoring.
*Static Tools*	Tools that do not involve active user input. Contains focused information from the full CPGs, formatted for single purpose use (e.g. Appendix PDF, 1 to 2-page PDF tools, slide decks, patient handouts).

**Table 2:** DC Guideline website components and descriptions

*Home Page*	The page the website starts on when entered through the main URL.
*Home Page Quick Buttons*	Graphical icons on the home page directly linking to 3 key messages and 8 interactive tools.
*Left-hand menu bar*	Navigation bar that stays on the left side of the website on all pages. Links are subheadings organized under 6 headings (e.g. “Guidelines”).
*Search*	Website search function accessed through the website’s top left corner. Defaults to “Exact Phrase” search. Other options available to switch to are “Any Word” and “All Words” search.
*Home Button*	Diabetes Canada logo button on top right corner links back to the Home Page.
*Tools & Resource Gallery*	Two separate pages for HCPs or for patients, accessible from the left-hand menu bar links. Page contains comprehensive list of links to tools and resources on the website, divided under headings. Each heading is an accordion menu (i.e. heading is visible but list of links is only visible after clicking on the heading).

### Participant and setting

We recruited clinicians from four professions frequently involved in diabetes care: family physicians, nurses, pharmacists, and dieticians. Investigators performed convenience sampling by emailing their own personal and professional network of health care providers from across Canada. From there, snowball sampling was used to elicit further contacts [15]. In order to ensure an adequate number of dieticians were represented, the publicly available Dieticians of Canada registry was utilized to cold email potential participants [16]. All participants were assigned to a randomly generated numeric code for data collection.

### Outcomes

We assessed the following outcomes for the usability tasks:

#### Task success rate

Successful completion was defined based on whether the response given by the user matched an acceptable answer from the investigators’ answer key.

#### Task completion time

The time from the initiation of first movement on the screen after instructions have been given until either a) a complete answer is given or b) the user asks to move on to the next task. If the user made further attempts for exploration purposes after completing the task initially, the extra time was not counted.

#### Clicks per task

The total number of clicks users made until the task was completed or aborted.

#### Resource used to complete task

The type of resource on the CPG website (full guidelines, a static tool, or interactive tool) participants used to complete a task successfully or unsuccessfully. If an attempt was aborted, the last page the participant landed on was considered to be the resource accessed.

#### Path

The links and pages accessed by the user, in the order in which they accessed them, from the beginning of a task until the task was completed or aborted.

#### Start of path

The section of the website (quick buttons on the home page, the links of the navigation menu, or the search function) that lead the users to the resource used to complete task. As users could explore multiple resources before arriving at the final answer, we defined the start of the path as being the most proximal to the final answer. For example, if a user followed the path [home page button ➔ page A ➔ back ➔ navigation menu link ➔ page B], the start of the path for the task would be navigation menu, as it is most proximal to page B, the resource used to complete the task.

#### Search attempts and success rate

The number of times users tried to use the website’s built-in search function and whether they succeeded in arriving at the correct resource.

#### Error type

Scheme was developed in data analysis (described below) to categorize unsuccessful task attempts into distinct error types.

### Data collection

#### Demographic and practice characteristics questionnaire

Participants completed a sixteen-item electronic questionnaire prior to usability testing sessions via email. This included questions about patient’s age, practice type and setting, and prior familiarity with the Diabetes Canada CPG website (Additional File 1).

#### Usability testing

Usability testing was conducted remotely over the internet using proprietary video conferencing software (GoToMeeting 2019 version) [17]. The software allowed participants to share their computer screen and communicate by audio with the investigator (WW). Sessions were approximately 1 hour in length. The sessions were recorded in video format with corresponding audio.

Each participant completed eight usability testing tasks in random order. Instructions for the tasks was provided in writing using the text chat feature of the conferencing software.

Participants vocalized their thought process aloud using the “Think Aloud” methodology [18, 19].

Audio was transcribed to text in non-strict verbatim format with filled pauses (i.e. “um”, repeated words, and stutters) removed. Video was used to transcribe the pathways participants took from the start to completion of each task into text format.

#### Tasks

The eight usability tasks (Table 3 and Additional File 2) were developed to reflect commonly encountered clinical situations in the diagnosis and treatment of diabetes. Some of these scenarios were used in the usability testing of the previous iteration of the Diabetes Canada CPG website. We included scenarios that could be answered using five of the six most accessed tools on the previous iteration of the Diabetes Canada website, in order to ensure the most popular tools were being assessed [11]. Five other clinicians uninvolved in the study were asked for input to further refine the tasks.

**Table 3:** Usability testing tasks. T2DM = type 2 diabetes mellitus

Task #	Task Type	Topic Assessed	Instruction (details omitted)
1	Information retrieval	Screening and diagnosis	Does she need any further testing?
2		Vascular protection	Does he need any medications for vascular protection?
3		Pharmacologic therapy for T2DM	What specific pharmacologic options would you consider?
4		Self-monitoring blood glucose	How often should he self-monitor his blood glucose?
7		Diabetes and pregnancy	What do the latest guidelines recommend about her pharmacologic therapy?
5	Resource retrieval	Stroke prevention	Can you provide her with a resource on ways to prevent a stroke?
6		Diabetes and mental health	Please retrieve the video resource on diabetes and mental health.
8		Safe driving	Please access a tool that you could use to counsel him on safe driving.

The usability tasks were divided into two types: information retrieval and resource retrieval. A previous study of the 2013 version of the website found that users likely entered the website to use a specific resource or to answer a specific clinical question [11].

The five information retrieval tasks asked participants to find the answer to a clinical question in a hypothetical clinical scenario (Table 3). For example, a task would describe a patient with diabetes and ask the clinician how often they should monitor their blood glucose. Resource retrieval tasks asked participants to locate a tool on the website, such as a patient handout. An answer key was developed with predetermined acceptable correct answers. All tasks could be completed using at least two resources on the website (e.g. full guideline chapter and quick reference guide).

#### Semi-structured interviews

Immediately after completing usability tasks, participants were interviewed to answer thirteen open-ended questions pertaining to the strengths and weaknesses of the website. The questions specifically asked for feedback on the website content as well as the format (see Additional File 3 for interview guide).

### Data analysis

#### Demographic and practice characteristics questionnaire

Results were entered into a spreadsheet and descriptive statistics performed.

#### Usability testing

Transcripts and path texts were analyzed to extract the outcomes defined above for the usability tasks. Descriptive statistics was performed.

One investigator (WW) reviewed the recordings and think-aloud transcripts of all unsuccessful attempts and developed a preliminary coding scheme for type of error made (Table 4). This was then applied by a second investigator (DC) independently to recode the errors. All disagreements were reviewed and settled by discussion.

**Table 4:** Type of errors

Type of Error	Description
Resource	User selected the wrong resource to complete the task
Interpretation	User gave incorrect answer despite finding the correct resource
Aborted	User was unable to find the answer and verbalized “giving up”
User	User did not follow instructions correctly

#### Semi-structured interviews

We performed qualitative content analysis of the transcripts of the semi-structured interviews using grounded theory, similar to previous studies in usability [20]. Two investigators (WW, DC) independently performed open coding of the transcripts to derive the initial themes, sorted under the categories of positive comments, negative comments, and suggestions for improvement [15]. We then performed axial coding of these initial themes to identify strengths and weaknesses of the website in terms of content and format.

## Results

### User characteristics

Fourteen clinicians participated in the study (Table 5). Most of the participants were young (age 20–39) and female. All participants had heard of the 2018 DC CPG website and 12 of 14 (85.7%) had used the website previously.

**Table 5:** User characteristics

Characteristics	*N* = 14	%
Profession		
Family physician	3	21.4
Nurse	4	28.6
Pharmacist	4	28.6
Dietician	3	21.4
Certified Diabetes Educator (*N* = 11)		
Yes	6	54.5
No	5	45.5
Sex at birth		
Female	9	64.3
Male	5	35.7
Age		
20–39	9	64.3
40–59	5	35.7
Type of Practice		
Academic	8	57.1
Community	2	14.3
Both	4	28.6
Heard of the 2018 DC Guidelines Website	14	100
Used the 2018 DC Guidelines Website	12	85.7
Accessed the 2018 DC Guidelines by		
Computer	13	92.9
Mobile	2	14.3

### Success rate, completion time, clicks taken

Overall, users had a 79% success rate across all tasks and used 144 (± 152) seconds and 4.6 (± 3.9) clicks per task (Table 6). Resource retrieval tasks had a higher success rate than information retrieval tasks.

**Table 6:** Success rate, time taken, and clicks for usability tasks

Tasks	Success rate (%)	Time taken (sec ± SD)	Clicks (± SD)
All tasks (*n* = 112)	**79**	**144 ± 152**	**4.6 ± 3.9**
Information retrieval tasks (*n* = 70)	**71**	**160 ± 86**	**4.0 ± 4.2**
1: Screening and diagnosis	57	86 ± 41	3.1 ± 1.3
2: Vascular protection	100	101 ± 33	2.9 ± 2.8
3: Pharmacologic therapy for T2DM	71	166 ± 112	3.2 ± 3.2
4: Self-monitoring of blood glucose	86	120 ± 72	2.8 ± 1.4
7: Diabetes and pregnancy	43	325 ± 247	8.1 ± 6.9
Resource retrieval tasks (*n* = 42)	**93**	**117 ± 134**	**5.5 ± 3.4**
5: Stroke prevention	86	151 ± 96	6.7 ± 3.7
6: Diabetes and mental health	93	112 ± 93	5.4 ± 3.6
8: Safe driving	100	88 ± 82	4.4 ± 2.5

Task 7 had the lowest completion rate of 43%. Users also spent the longest time and most clicks on this task. Task 1 had the second lowest completion rate of 57% but was the shortest task at 86 (± 41) seconds.

### Resource used and path taken

Interactive tools were more frequently used to complete tasks successfully than the full guidelines and static tools (Table 7). In unsuccessful attempts, the three types of resources were used about equally. The Quick Reference Guide was the most frequently used among static tools.

**Table 7:** Resource accessed when completing information retrieval tasks

Resource	Total # of Times Resource Accessed (% Total Tasks)	# of Times Accessed when Successful (% Successful Tasks)	# of Times Accessed when Unsuccessful (% Unsuccessful Tasks)
2018 Full Guidelines	12 (17%)	5 (10%)	7 (35%)
Interactive Tools	37 (53%)	30 (60%)	7 (35%)
Quick Reference Guide	15 (21%)	10 (20%)	5 (25%)
Static Tools			
Appendix	3 (4%)	3 (6%)	0 (0%)
Slides	1 (1%)	1 (2%)	0 (0%)
PDF	2 (3%)	1 (2%)	1 (5%)

The homepage quick buttons leading directly to interactive tools were almost as frequently the start of final paths as links on the navigation bar (Table 8).

**Table 8:** Start of path when completing information retrieval tasks (*n* = 70)

Start of paths	Number of times resource was start of path (% of total start of path)
Homepage Quick Buttons	32 (45%)
Lefthand Menu/Navigation Bar	38 (54%)
Search	0 (0%)

### Search function

Although the search function was not used to complete information retrieval tasks (Table 8), some users did use search unsuccessfully before changing their strategy, while others used it to complete resource retrieval tasks. In total, the website’s built-in search was accessed 13 times and successfully found an answer or resource in 2 of the 13 attempts (15.4%).

### Errors

Interpretation error accounted for 48% of total errors (Table 9). Task 1 alone accounted for over half of these, with all six unsuccessful attempts being interpretation errors. They were made by three of four users that accessed the quick reference guide, containing an algorithm, and three of nine users that accessed the screening interactive tool. Users gave up most frequently (3 of 8 instances) in task 7 on pregnancy and diabetes.

**Table 9:** Types of errors committed by task

	Interpretation Error	Resource Selection Error	User Error	Attempt Aborted
Task 1 (*N* = 6)	–	6 (100%)	–	–
Task 3 (*N* = 4)	–	2 (50%)	1 (25%)	1 (25%)
Task 4 (*N* = 2)	–	1 (50%)	1 (50%)	–
Task 5 (*N* = 2)	2 (100%)	–	–	–
Task 6 (*N* = 1)	–	–	–	1 (100%)
Task 7 (*N* = 8)	–	2 (25%)	3 (38%)	3 (38%)
**Total (*N* = 23**)	**2 (9%)**	**11 (48%)**	**5 (22%)**	**5 (22%)**

(Task 2 and Task 8 had perfect completion and are not shown on this table.)

### Content-related themes

The top strengths and weaknesses pertaining to website content are shown in Table 10.

**Table 10:** Top strengths and weaknesses of website content identified through content analysis and their representative quotes

Content Themes	Description	Representative Quote
Strength		
Interactive tools (10)	An interactive tool is particularly useful	“[…] having them […] spit out a recommendation I found that really helpful as well as quick.” (User K, Dietician)
Static tools (9)	A static tool is particularly useful	I usually go to the appendices, and I really like this renal [...] This is probably one that I go through on a daily basis because I just cannot remember, memorize this. So that was really good. (User C, Physician)
Quick reference guide (5)	Quick reference guide is particularly useful	“For most of the scenarios and in clinical practice, for all the iterations of this quick reference guide, I’ve found really […] useful.” (User F, Pharmacist)
Comprehensiveness (4)	Information on the CPG website is comprehensive	“[…] just the wealth of information is very impressive […] if I do need to find out anything […] I can also go to this website.” (User M, Nurse)
Weakness		
Full guidelines (7)	Full guidelines are not as easy to use as tools and summaries	“Obviously, the guidelines need to be there but […] I found it less helpful to have to read through the guidelines […] to find something, rather than having […] a table already […] set out for me on […] specific recommendation that I’m looking for.” (User K, Dietician)
Not enough content (5)	Content is sparse or not available for some topics	It’s really tough to find everything you want. [...] one of the things people have a lot of trouble with is exercise […] when you talk about dealing with exercise and making adjustments to insulin [...] I usually suggest they go to a different website for that. (User J, Pharmacist)
Medical jargon (4)	Medical jargon can be confusing for patient-aimed resources	“How does a person living with diabetes come on here and decide oh, is my heart “micro or macro” right [...] This is not at patient speak language in any way, shape, or form” (User D, Nurse)
Not enough visual aids (4)	More visual aids (e.g. pictures, flow charts) would be useful	“I think there could be a few more flowcharts and things […] that kind of includes more of the advanced conditions, if that makes sense.” (User H, Dietician)

Users showed a preference for interactive tools, static tools, and quick reference guides, while the full guidelines text was perceived as less useful. Users liked that the interactive tools were “quick” in generating answers.

Several themes addressed the presentation of information within the content. Users wanted to see more visual representations of content, such as tables and flowcharts. Some users pointed out there was too much medical jargon on the website, which could make it less useful for patients.

Users had mixed opinions about the breadth of content. Several users praised the website for being comprehensive, but similar number of users thought content was sparse in certain clinical areas.

### Format of the website

The top strengths and weaknesses pertaining to website format are shown in Table 11.

**Table 11:** Top strengths and weaknesses of website format identified through content analysis and their representative quotes

Format Themes	Description	Representative Quote
Strengths		
Navigation bar (9)	Navigation bar on the left hand side facilitates use of the website	“I […] like the bar at the side that doesn’t change […] So I can always jump back to different categories.” (User G, Physician)
Quick links on home page (6)	Direct link icons to tools on the home page facilitates navigatio	“I liked the fact that when you are on the home page, you immediately see the clinical decision support tools. That’s where my focus is when I’m on the home page. So it’s useful for me […] when I want to answer one question more quickly, as opposed to looking through the guidelines.” (User I, Pharmacist)
Intuitiveness (5)	The website is easy or intuitive to use	“[…] if I were to be presented with […] resources that I wanted to use […] I think I would be able to easily go into the website and find what I was looking for within 20 seconds, maybe 30 seconds. So it was pretty simple to navigate overall. (User N, Dietician)
Headings (4)	Headings used to divide content on the website is effective	“[…] having all the tools be in one area and […] sorted into different categories based off if they’re more diagnosis or management, or certain populations, so that it’s a little bit easier to navigate through and find what you want that way.” (User G, Physician)
Redundancy in navigation (4)	There are multiple ways to access the same content	“I like that there’s a lot of overlap […] for example that drive safe was under hypoglycemia and it’s [also] under driving so it’s easy to find things that way.” (User A, Pharmacist)

Navigation elements were the most highly rated aspects of the website design. Most users found the left-hand navigation bar useful. Almost half of users found the links to the tools on the main page helpful. Being able to access the same information in multiple ways was also seen as a positive.

Many users still found it difficult to find information. Links that were formatted as lists, rather than as buttons, were challenging to use for most users. About a third of users thought the search function did not work well.

## Discussion

### Overall usability

Within the context of our chosen tasks, the Diabetes Canada CPG website had good usability with a task success rate for both information and resource retrieval of 79%. Users brought up many positive qualities of the website in the interview, including praise for the individual content on the website as well as design elements. A third of users praised the website for being intuitive.

### Effective content

The different versions of guideline content on the Diabetes Canada CPG website facilitated task completion. For the information-retrieval tasks, the majority of successful attempts (90%) were completed using the interactive tools, static tools, or quick reference guide, rather than the full guideline chapters. Users also considered the alternate versions to be strengths of the website, while full guideline chapters were perceived to be less useful.

The semi-structured interview elicited “Comprehensiveness” as a strength and “Not enough content” as a weakness of our website, which may seem contrary to the barriers of time and information overload. One explanation is that CPGs can have serve other purposes, such as for dedicated learning outside of clinical care, in which there are less time constraints. Guideline use can be improved by having alternate versions for different purposes [7]. Clinicians have significant point-of-care information needs, raising 0.18 to 1.5 questions on the average patient, most commonly regarding treatment and diagnosis [21]. However, time and information overload are important barriers to seeking information [21–23]. Even at the point-of-care, clinicians can have different preferences regarding volume of information, with “competing demands of brevity and comprehensiveness” [20, 24]. While full text guideline documents are more comprehensive, alternate versions contain targeted information which can be utilized more quickly at point-of-care. Thus, creating websites with multiple versions of the guidelines in one place gives users the choice of how and how much CPG information they want to use, and may ultimately improve uptake.

Users also wanted to see more visual content such as tables and algorithms on the website. Visual presentation can enhance information delivery and has been recommended by multiple authors [3, 25, 26]. Interestingly, we found that while visual presentation may speed up information delivery, it does not guarantee accuracy: task 1 took users the least time to complete, yet it was the task with the second most errors. Usability testing should be considered for elements like algorithms, to ensure accurate information delivery.

### Effective format

Existing recommendations for CPG format include using boxes to highlight recommendations, algorithms and tables, different versions, and layering [5, 6, 26, 27]. However, little recommendations exist in the CPG literature pertaining to web design.

Most users brought up the fixed navigation bar on the website as a strength, as it allowed them to jump around different sections without using the back button. Many users also liked the quick buttons on the home page that linked to the interactive tools. In contrast, organization of links in other parts of the website were rated poorly as was the search function, which performed poorly, with only 15% of attempts generating the desired result. A major weakness of the search function we identified was the default search mode of “exact phrase” for search terms. This could lead to omission of desired results if the phrase is worded differently on the webpage or if the user makes a spelling mistake. The algorithm may also be flawed, as we sometimes observed less relevant webpages containing the search terms prioritized over the more relevant webpage in the results. The World Wide Web Consortium’s guidelines on web accessibility recommends implementing search functions that can generate results that account for spelling errors, different endings on search terms (stemming), and use of synonyms [28]. It also recommends using techniques like meta tags to optimize results. Our next steps for improving the website includes re-designing the search function to reflect these recommendations.

In our study, the average amount of time taken to complete information retrieval tasks was only 160 seconds. This is comparable to the amount of time actual users spent per session on the previous iteration of the Diabetes Canada CPG website (180 seconds) and less than the amount of time internal medicine physicians have been observed searching online sources while working (252 seconds) [11, 29]. When a task took much longer to complete (task 7 at 352 seconds), it resulted in the lowest success rate (43%), with some users giving up after not finding information quickly, highlighting the importance of intuitive navigation.

The strengths and weaknesses identified in our study of a CPG website are echoed in existing website design guidelines not specific to clinician use. For instance, the use of navigation menus (left-hand positioning specifically for primary menus) is recommended in the Research-Based Web Design & Usability Guidelines published by the U.S. Department of Health and Human Services (HHS) [30]. Both the HHS and International Organization for Standardization (ISO) guidelines recommend allowing access to important options on the home page and providing an effective search function [30, 31].

### Recommendations for CPG website design

Based on our findings and literature review, we have synthesized a list of recommendations for guideline developers and disseminators (Table 12) that can be adapted or added to existing guideline implementation planning checklists and tools [32]. Incorporating web design recommendations in the assessment of CPG websites could lead to improvements in their usability and uptake.

**Table 12:** Recommendations for the design of Clinical Practice Guideline websites based on our findings and literature review

Recommendation	Rationale/Description
*During website design:*	
Provide different versions (e.g. interactive tools, static tools, quick reference guide, full chapter) of guideline content on the website.	Different versions facilitate task completion (e.g. answering a clinical question at the point-of-care) and fulfill different purposes (clinical care vs self-study)
Emphasize visual content (e.g. tables, algorithms)	Visual content speeds up information delivery.
Offer multiple navigation options (e.g. left-sided navigation bar, quick buttons on home page, search function).	Users appreciate having multiple methods to get to the desired information. Navigation bar, quick buttons on the home page and a robust search function enables more efficient use.
Use intuitive grouping of resources, and avoid long lists of links under broad headings	Using categories that mimic a user’s needs (e.g. management) facilitates resource location and minimizes user search fatigue.
*After website design (iterative refinement):*	
Conduct usability testing of the guideline website and its subcomponents (including algorithm testing)	Usability testing and iterative refinement optimizes task completion, user satisfaction and guideline uptake, and ensures accurate information delivery.

### Study limitations & strengths

Limitations include convenience sampling of participants, lack of iterative testing, and the inability of usability tasks to completely replicate real-world use of the website. Our study demographic overrepresented younger, female users (64% younger than age 40, 64% female) and experienced users. This may have affected some of the study results, although we know from real-world data of our website that the actual end users also skew towards younger, female users (64% younger than age 44, 70% female). Younger users are more likely to be comfortable with using technology and may be more adept at completing tasks. Sex has not been found to influence user experience of websites in other studies [33]. Previous experience may have led to better completion rate and speed, but studies show that experience affects these metrics minimally when web designs are highly effective [34]. Strengths of the study include the variety of usability tasks chosen, inclusion of participants with differing clinical backgrounds, mixed-methods design, and and independent coders.

## Conclusion

Multiple versions of CPGs (e.g. interactive tools, static tools, summaries) can be used to answer clinical questions more quickly. Usability testing can be used to identify previously unknown issues with CPG content. Effective web design should be assessed in the creation of CPG website.

## Supplementary Information

**Additional file 1.** Baseline questionnaire.

**Additional file 2.** Task and accepted answers.

**Additional file 3.** Semi-structured interview guide for usability testing.

## Data Availability

All data generated or analysed during this study are included in this published article [and its supplementary information files].

## Abbreviations

CPG: Clinical practice guidelines
DC: Diabetes Canada
HCPs: Health Care Providers
SMBG: Self-Monitoring of Blood Glucose
