# Supplementary material for: Effective web-based clinical practice guidelines resources: recommendations from a mixed methods usability study
Source: BMC Prim Care. 2023 Jan 24;24:29. doi: 10.1186/s12875-023-01974-1 (PMC9872348; doi:10.1186/s12875-023-01974-1)
Supplement: Supplementary file 1 — Additional file 1. Baseline questionnaire. [file 12875_2023_1974_MOESM1_ESM.docx]

**Additional File 1: Baseline questionnaire**

# Baseline questionnaire for DC Website Usability Testing – for providers

Study ID number: ____

Thank you for agreeing to participate in this study. Your input will help develop and refine tools that will help people with diabetes and health care providers decide on goals of care.

Participation in any research study is voluntary. You may skip any of the questions you do not feel comfortable in answering. These questions are designed to ensure that we have a variety of people giving us their opinion about these diabetes tools.

Please answer the following questions about yourself. Your answers to these questions will be kept completely confidential.

1. What is your gender
   - Male
   - Female
2. What is your age now?
   - Less than 20 years old
   - 20 to 39 years old
   - 40 to 59 years old
   - 60 to 79 years old
   - More than 80 years old
3. What year did you start clinical practice? ______________
4. Is English your first language?
   - Yes
   - No
5. What type of health care provider are you?
   - Family physician
   - Nurse
   - Dietitian
   - Pharmacist
   - Other: ____________________
6. Are you a certified diabetes educator?
   - Yes
   - No
7. How are you remunerated?
   - Salary
   - Fee for service
   - Combination
8. What setting do you work in?
   - Academic
   - Community
   - Both

How comfortable to you feel about managing a person’s diabetes?

- - Very comfortable
  - Comfortable
  - Neutral
  - Uncomfortable
  - Very uncomfortable

1. In an average week, how many patients do you see? ____________ [free text]
2. How many of these patients have diabetes (including type 1 diabetes, type 2 diabetes or gestational diabetes)? ____________ [free text]
3. Have you ever heard of the Diabetes Canada 2018 Guidelines website?
   - Yes
   - No
4. Have you ever accessed the Diabetes Canada 2018 Guidelines website?
   - Yes
   - No
5. Which section(s) of the Diabetes Canada 2018 Guidelines website have you accessed?
   - Guidelines
   - Key Messages
   - For Healthcare Providers
   - Tools for People with Diabetes
   - Other Languages
6. For what percentage of patients do you currently utilize the Diabetes Canada 2018 Guidelines website in facilitating a patient’s health care?
   - Never
   - 0.1-25%
   - 25-50%
   - 50-75%
   - 75-100%
7. How do you primarily access for the Diabetes Canada 2018 Guidelines website?
   - Desktop or notebook computer
   - Tablet
   - Mobile phone
   - Not applicable
