# Supplementary material for: Effective web-based clinical practice guidelines resources: recommendations from a mixed methods usability study
Source: BMC Prim Care. 2023 Jan 24;24:29. doi: 10.1186/s12875-023-01974-1 (PMC9872348; doi:10.1186/s12875-023-01974-1)
Supplement: Supplementary file 2 — Additional file 2. Task and accepted answers. [file 12875_2023_1974_MOESM2_ESM.docx]

**Additional File 2: Tasks and accepted answers**

| Task | Instructions | Accepted Answers |
| --- | --- | --- |
| 1 | Patient A is a 40-year-old woman who has a fasting plasma glucose of 5.7 mmol/L. Her father has type 2 diabetes. Does she need any further testing? | - Rescreen more frequently (q 6-12 months) +/- consider A1C |
| 2 | Patient B is a 52-year-old man who has had type 2 diabetes since 1994. He is well-controlled with an A1C of 6.9% on 3 non-insulin agents. He takes no other medications. He has no history of microvascular or macrovascular disease, and his urine ACR is undetectable. His LDL is 2.2. Does he need any medications for vascular protection? | - Start statin therapy |
| 3 | Patient C is a 36-year-old woman who is newly diagnosed with type 2 diabetes. She also has CHF, metabolic bone disease, and a history of pancreatitis. Her GFR is 62. Her A1C is 9.5% and she has problems affording medications. What specific pharmacologic options would you consider? | - Healthy intervention + metformin + consider sulfonylurea - Acceptable if user states will use the table to discuss with patient what the priorities are using the pharmacologic therapy table - As CHF is not considered cardiovascular disease, SGLT2 inhibitors which are not indicated and expensive was counted as incorrect |
| 4 | Patient D is a 44-year-old man who has a 5-year history of type 2 diabetes. He is taking metformin, gliclazide, and sitagliptin with no insulin. His A1C is 9.9% and he self-monitors his blood glucose (SMBG) very infrequently. How often should he self-monitor his blood glucose? | - SMGB >=1 or >=2 depending on tool used |
| 5 | Patient E is an 18-year-old woman with type 1 diabetes. Her mother also has type 1 diabetes and just had a stroke. The patient is very fearful of getting a stroke. Can you provide her with some resources from the DC guidelines website on ways to prevent a stroke? | - “ABCDESSS of Staying healthy with diabetes” PDF - “Are there medications that can reduce my risk of heart disease and stroke?” Interactive tool - “Heart disease and Stroke” PDF - “What medications should I be taking to protect myself from heart disease and stroke?” PDF - Any other tool or resource that provides some information for patient on ways to stay healthy |
| 6 | Patient F is a 22-year-old man with type 1 diabetes. He also has been recently diagnosed with schizophrenia. You are reviewing your knowledge about diabetes and mental health. Please retrieve the video resource on diabetes and mental health and watch the first 20 seconds of this video. | - Locate the correct video |
| 7 | Patient G is a 32-year-old woman with type 2 diabetes. She is taking metformin, insulin, and atorvastatin. She just found out that she is pregnant. She has an appointment to see her endocrinologist for further management in 3 days. What do the latest DC guidelines recommend about her pharmacologic therapy? | - Continue insulin, can continue metformin (this was not strictly scored), stop atorvastatin |
| 8 | Patient H is a 46-year-old man with type 2 diabetes. He takes metformin and insulin and occasionally experiences hypoglycaemia at home. Please access a tool on the DC guidelines website that you could use to counsel him on safe driving. | - “Drive Safe with Diabetes” PDF - Quick Reference Guide Page 6 - Appendix 10 – “Diabetes and Driving Assessment Form” |
