# Supplementary material for: Effective web-based clinical practice guidelines resources: recommendations from a mixed methods usability study
Source: BMC Prim Care. 2023 Jan 24;24:29. doi: 10.1186/s12875-023-01974-1 (PMC9872348; doi:10.1186/s12875-023-01974-1)
Supplement: Supplementary file 3 — Additional file 3. Semi-structured interview guide for usability testing. [file 12875_2023_1974_MOESM3_ESM.docx]

**Additional File 3: Semi-structured interview guide for usability testing**

Now we will discuss a few questions relating to the website:

1. What are some general strengths of this website?
2. What are some general weaknesses of this website?
3. What content did you find particularly helpful?
4. What content did you find was not helpful?
5. Is there any content missing on the website?
6. What did you think of the format of the website?
7. Which aspects of the format did you find particularly helpful?
8. Which aspects of the format did you find not helpful?
9. If you could change just one thing about the tools on the website, what would it be?
10. Inputting information: How do you feel about entering data in the website? Were there any problems with this process?
11. Please let us know any final comments that you have on anything good, bad or confusing that will enable to identify problems and improve the website.
12. Do you have anything else to add?
13. Do you have any questions for me?
